# Grundlagen der Nierenpathologie für Pathologen – Teil 2: Nichtentzündliche Veränderungen

**DOI:** 10.1007/s00292-023-01204-6

**Published:** 2023-06-27

**Authors:** Ariana Gaspert, Maike Büttner-Herold, Kerstin Amann

**Affiliations:** 1 Abteilung für Nephropathologie, Institut für Pathologie und Molekularpathologie, Universitätsspital Zürich, Schmelzbergstr. 12, 8091 Zürich, Schweiz; 2 Abteilung für Nephropathologie, Institut für Pathologie, Friedrich-Alexander Universität Erlangen-Nürnberg und Universitätsklinikum Erlangen, Erlangen, Deutschland

**Keywords:** Nephropathie, Hypertension, Diabetische Nephropathie, Thrombotische Mikroangiopathie, Differenzialdiagnose, Nephropathy, Hypertension, Diabetic nephropathy, Thrombotic microangiopathy, Differential diagnosis

## Abstract

Die Nierenbiopsiediagnostik bei medizinisch indizierten Biopsien und Nierentransplantatbiopsien wird überwiegend in Zentren mit ausgebildeten Nephropathologen betrieben. Veränderungen im nichtneoplastischen Nierenparenchym bei tumorbedingten Nephrektomien, insbesondere nichtentzündliche, ischämisch und vaskulär bedingte Veränderungen oder diabetische Nephropathie, können bei Patienten mit lokalisiertem Nierenkarzinomen und gutem tumorassoziiertem Überleben prognostisch von größerer Bedeutung sein als das Tumorleiden an sich. In diesem Teil zu den Grundlagen der Nierenpathologie für Pathologen sollen die häufigsten nichtentzündlichen Nierenerkrankungen im vaskulären, glomerulären und tubulointerstitiellen Kompartiment beleuchtet werden.

## Abstract (en)

The evaluation of kidney biopsies for specific renal diseases or kidney transplant biopsies is mainly restricted to specialized centers. Lesions in nonneoplastic renal tissue in partial nephrectomies or nephrectomies due to renal tumors, especially noninflammatory, ischemic, vascular changes or diabetic nephropathy can be of greater prognostic significance than the tumor itself in patients with a localized tumor and good tumor-associated survival. In this part of basic nephropathology for pathologists, the most common noninflammatory lesions of the vascular, glomerular and tubulo-interstitial compartment are discussed.

## Lernziele

Nach Lektüre dieses Beitrags …

- kennen Sie die Hauptkompartimente der Niere;
- sind Ihnen die wichtigsten nichtentzündlichen Erkrankungen der Niere bekannt;
- können Sie wichtige Erkrankungen in Nephrektomiepräparaten erkennen, die nebst Tumoren in der Niere für den Patienten prognostisch relevant sind;

## Hintergrund

Diabetes, Hypertonie, Adipositas, Rauchen und Altern sind unabhängige Risikofaktoren für das Entstehen von Nierenzellkarzinomen [1]. Es ist deshalb nicht erstaunlich, dass in einer Studie nur 10 % der **Tumornephrektomien**

Tumornephrektomien

unauffälliges Nierenparenchym und Gefäße zeigten und in bis zu 28 % der Fälle zwar unauffälliges Parenchym, aber Arteriolo- oder Arteriosklerose mindestens geringen Ausmaßes oder aber auch stark ausgeprägt gefunden wurde [2]. Mehr als 60 % der Nephrektomien in dieser Studie wiesen **pathologische Veränderungen**

pathologische Veränderungen

auf, meistens aufgrund vaskulärer Erkrankungen oder Diabetes mellitus [2]. Diese Patienten haben ein Risiko, eine **progressive Nierenerkrankung**

progressive Nierenerkrankung

nach Nephrektomie zu entwickeln [2]. Diese klinisch relevanten Nierenerkrankungen können im **tumorfreien Nierenparenchym**

tumorfreien Nierenparenchym

bei Nephrektomien und teilweise bei genügend Parenchym auch in Nierenteilexzidaten diagnostiziert werden. Wenn der Beurteilung des nichtneoplastischen Nierenparenchyms keine Aufmerksamkeit geschenkt wird, können koinzidentelle Nierenerkrankungen verpasst werden, was in einer Studie in bis zu 88 % der Fälle beobachtet wurde [3]. Andere Erkrankungen als Diabetes und Hypertonie, die mit dem Tumor oder der Therapie assoziiert sein können, sind z. B. thrombotische Mikroangiopathie, Amyloidose, membranöse Glomerulonephritis, IgA-Nephropathie, membranoproliferative Glomerulonephritis, pauci-immune Glomerulonephritis, fokal segmentale Glomerulosklerose, Minimal-change-Glomerulopathie, akute interstitielle Nephritis und xanthogranulomatöse Pyelonephritis [4]. Da **chronische Nierenerkrankungen**

chronische Nierenerkrankungen

bei klinisch lokalisierten Nierentumoren häufig bezüglich Überleben von Patienten und Patienten, die mit Endstadiumnieren an der Dialyse eine bedeutend **niedrigere Lebenserwartung**

niedrigere Lebenserwartung

haben, ausschlaggebender sind als Nierenzellkarzinompatienten im Stadium I, II oder III, gibt dies Pathologen und Nephrologen die Möglichkeit, die Diagnostik und das Management einer chronischen Nierenerkrankung zu verbessern [1].

### Einfluss des Alterns

Das Altern ist assoziiert mit signifikanten Veränderungen der Struktur und Funktion der Niere, auch in Abwesenheit altersabhängiger Komorbiditäten und ohne Erhöhung des Risikos für eine terminale Niereninsuffizienz oder Mortalität [5]. Diese Veränderungen betreffen auf **mikrostruktureller Ebene**

mikrostruktureller Ebene

vor allem die Arteriosklerose, Arteriolosklerose, ischämisch alterierte Glomeruli, globale Glomerulosklerose, interstitielle Fibrose und Tubulusatrophie. Dies sollte bei der Beurteilung einer Nierenbiopsie, aber auch der Beurteilung des tumorfreien Nierenparenchyms berücksichtigt werden. Man sollte „gesundes Altern“, bzw. altersentsprechendes, unauffälliges Nierengewebe von „Altern mit **erwarteten Komorbiditäten**

erwarteten Komorbiditäten

“ (z. B. bei Patienten mit Hypertonie, Diabetes und Adipositas) und von „**chronischen Nierenkrankheiten**“

chronischen Nierenkrankheiten

(z. B. Diabetes mit IgA-Nephropathie) unterscheiden. In einer Studie an Nierenbiopsien von Nierenlebendspenden [6] wurde das normale Altern der Niere untersucht und die 95. Perzentile für die Anzahl vollständig **verödeter Glomeruli**

verödeter Glomeruli

als Funktion des Alters und der totalen Anzahl an Glomeruli eruiert. Für eine Biopsie mit 17–32 Glomeruli reichte die 95. Perzentile von 1 für 20-jährige bis 5,5 für 70-jährige Spender. Diese altersspezifischen Schwellenangaben für **Glomerulosklerose**

Glomerulosklerose

können insbesondere in Nierenbiopsien den Klinikern helfen, die Bedeutung der Glomerulosklerose einzuschätzen.

### Dokumentation von Veränderungen im nichtneoplastischen Parenchym und Färbemethoden

Gemäß Empfehlung des College of American Pathologists [7] sollten bei der Untersuchung von Resektaten von Nierenzellkarzinomen auch Veränderungen im nichtneoplastischen Parenchym berichtet werden. Als Empfehlung für ausreichendes Gewebe gilt das Vorhandensein von 5 mm nichtneoplastischen Nierenenparenchyms. In Fällen, in denen kein Nierenparenchym vorhanden ist, sollte zumindest angegeben sein, dass beurteilbares nichtneoplastisches Nierengewebe fehlt.

Von einem Paraffinblock sollte für die **Ersteinschätzung**

Ersteinschätzung

je ein Schnitt mit Hämatoxylin-Eosin(HE)-Färbung, Periodic-Acid-Schiff(PAS)-Reaktion und Elastika-van-Gieson(EVG)-Färbung (für kollagene und elastische Fasern) angefertigt werden. Gegebenenfalls können Spezialfärbungen wie Kongorot, Versilberung (Methenamin für Basalmembranen), eine Saures-Fuchsin-Orange-G(SFOG)-Färbung (Proteine, Kollagenfasern) oder immunhistochemische oder **Immunfluoreszenzuntersuchungen**

Immunfluoreszenzuntersuchungen

(Immunglobuline A, G, M sowie Komplementkomponenten C3 [gemeinsame Endstrecke] und C1q [klassischer Weg], Immunglobulinleichtketten [κ und λ] oder verschiedene Amyloidtypen) folgen.

In diesem 2. Teil der Grundlagen der Nierenpathologie für Pathologen werden die am häufigsten im Rahmen der Nierentumorchirurgie zu diagnostizierenden Nierenpathologien sowie, der Vollständigkeit halber, andere, nicht entzündliche Veränderungen im vaskulären, glomerulären und tubulointerstitiellen Kompartiment besprochen.

## Vaskuläres Kompartiment

**Figure 1:**
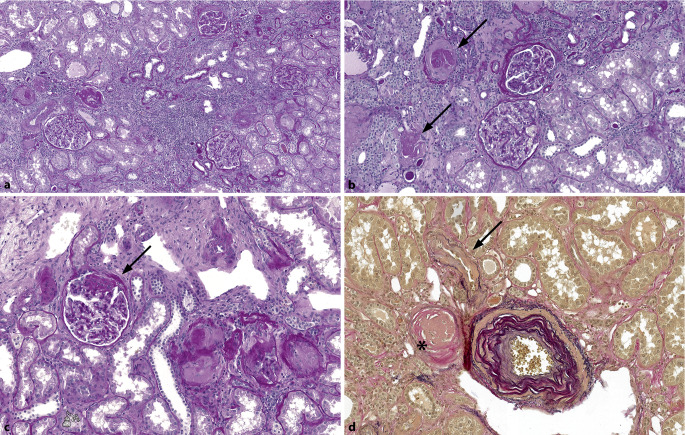

Mit arterieller Hypertonie assoziierte Veränderungen sind die häufigsten Befunde in Tumornephrektomien und entsprechen der Kombination unspezifischer Befunde wie globale Glomerulosklerose, interstitielle Fibrose, Tubulusatrophie, Arteriolo- und Arteriosklerose (Abb. 1; [1]). Als Synonyme zu **hypertensiver Nephropathie**

hypertensiver Nephropathie

werden manchmal die Begriffe Arterio‑/Arteriolonephrosklerose und hypertensive Nephrosklerose gebraucht [8]. Die Glomeruli können vermehrt mesangiale Matrix, Doppelkonturen der Basalmembran, eine segmentale und/oder globale Sklerose und glomeruläre Hypertrophie aufweisen. Es können **subkapsuläre Narben**

subkapsuläre Narben,

eine interstitielle Fibrose mit mononukleären Begleitinfiltraten sowie Tubulusatrophie und -hypertrophie auftreten [8]. Mittelgroße Arterien zeigen eine **Intimafibrose**

Intimafibrose,

vermehrt elastische Fasern (Fibroelastose), glattmuskuläre Hyperplasie und eine Verkleinerung des Lumens. Die Arteriolen zeigen subendothelial und im Verlauf auch in der Media eine hyaline **Arteriolosklerose**

Arteriolosklerose.

Arteriolo- und Arteriosklerose sind die häufigsten Veränderungen im Nierenparenchym. Generell ist man sich einig, dass beim Alter < 50 Jahren, die Anzahl vollständig verödeter Glomeruli < 10 % (wahrscheinlich auch < 5 %) sein sollte [2]. Bei > 50-jährigen ist die Unterscheidung zwischen „normal“ und „abnormal“ erschwert und bei > 10 % verödeter Glomeruli sollte man vorsichtig sein, die Veränderungen dem „normalen Alterungsprozess“ zuzuschreiben [2]. Ein anderer Ansatz für normale Alterungsprozesse ist z. B. 5 % globale Glomerulosklerose bei Kindern und jungen Erwachsenen sowie < ([Alter geteilt durch 2] minus 10)-% vollständig sklerosierte Glomeruli in älteren Individuen [9]. Der Nachweis von > 20 % globaler Glomerulosklerose ist ein **signifikanter Prädiktor**

signifikanter Prädiktor

eines wesentlichen Nierenfunktionsverlusts innerhalb von 6 Monaten nach chirurgischem Eingriff [1].

### Thrombotische Mikroangiopathie

Eine thrombotische Mikroangiopathie (TMA) kann im Kontext einer hypertensiven Krise oder schweren Hypertonie (früher auch maligne Hypertonie) vorkommen, aber auch im Rahmen vieler verschiedener anderer Erkrankungen, nicht zuletzt medikamentenassoziiert [10] z. B. im Rahmen der Therapie von Nierenzellkarzinomen. Typischerweise findet man bei TMA in Arteriolen und/oder Arterien **Fibrinthromben**

**Figure 2:**
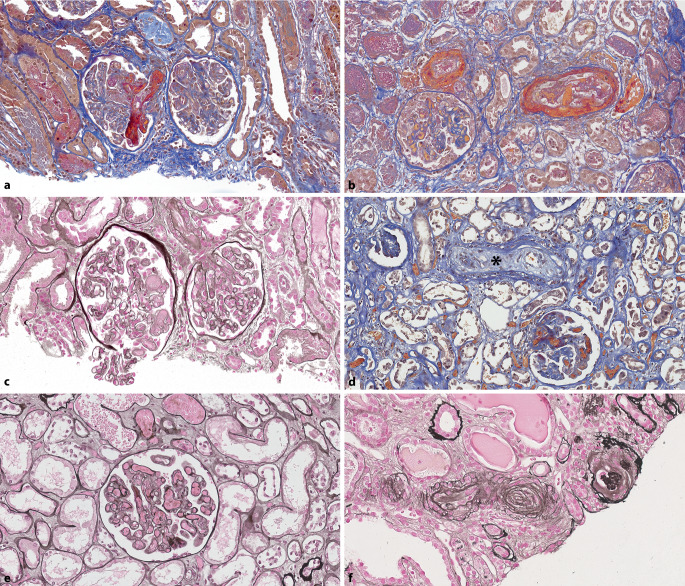

Fibrinthromben

im Lumen und/oder eine Fibrininsudation in der Gefäßwand, in Arterien mukoide Intimaveränderungen und/oder einen zwiebelschalenartigen Wandumbau und in den Glomeruli Fibrinthromben, ischämische Retraktion und/oder Doppelkonturen der Basalmembran sowie segmentale Sklerose (Abb. 2; [8]). Neben primären niereneigenen Erkrankungen finden sich in Tumornephrektomien immer wieder auch **sekundäre Veränderungen**

sekundäre Veränderungen

infolge der Tumortherapie, was grundsätzlich berücksichtigt und mitbeurteilt werden muss. Eine TMA wird bei verschiedenen klassischen **Chemotherapeutika**

Chemotherapeutika,

wie z. B. Gemcitabin, aber auch bei anderen Medikamentengruppen, wie z. B. Antikörper gegen VEGF („vascular endothelial growth factor“) und Tyrosinkinaserezeptorinhibitoren, beschrieben [11, 12]. Das morphologische Schädigungsmuster bei anti-VEGF-induzierter Mikroangiopathie ist sehr charakteristisch und kann Hinweise auf die zugrunde liegende Therapie geben. Differenzialdiagnostisch muss mittels Immunhistochemie und Elektronenmikroskopie lediglich eine membranoproliferative Glomerulonephritis ausgeschlossen werden. Alle anderen Schädigungsmuster sind für keines der Medikamente spezifisch und bedürfen immer einer engen klinikopathologischen Korrelation.

### Cholesterinembolien

**Figure 3:**
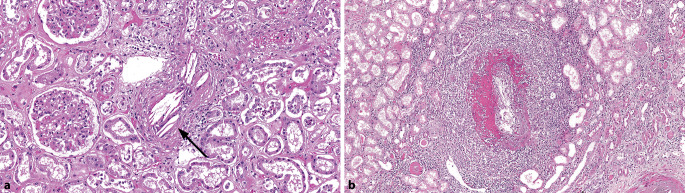

Cholesterinembolien (Abb. 3a) können insbesondere bei Patienten mit schwerer Atherosklerose spontan oder **iatrogen** nach Gefäßeingriffen, Kathetereingriffen, kardiopulmonaler Reanimation, Antikoagulanzientherapie

Antikoagulanzientherapie

oder Trauma entstehen [13].

### Wichtige Differenzialdiagnosen

Die **nekrotisierende Vaskulitis**

nekrotisierende Vaskulitis

(Abb. 3b) ist eine wichtige Differenzialdiagnose insbesondere gegenüber fibrinoiden Gefäßwandnekrosen bei thrombotischer Mikroangiopathie. Bei Vaskulitiden sind in der Niere meist auch die Glomeruli in Form von fibrinoiden Schlingennekrosen und extrakapillaren Proliferaten (sog. Halbmonden) involviert[14].

## Glomeruläres Kompartiment

Eine **mesangiale Matrixvermehrung**

mesangiale Matrixvermehrung

**Figure 4:**
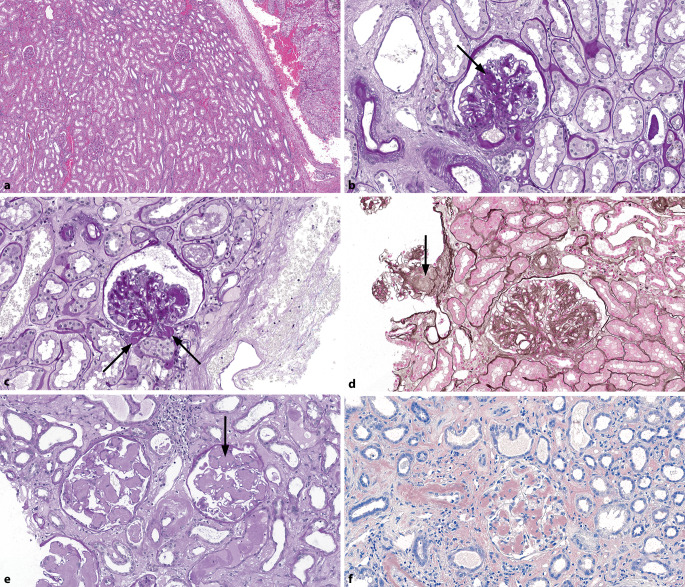

**, **auch *mesangiale Sklerose* genannt, ist durch Matrixablagerungen definiert, die die Breite von 2 Mesangiumkernen überschreiten [15] und auch nodulär imponieren können. Die häufigste Ursache einer nodulären Glomerulosklerose ist Diabetes mellitus (Abb. 4a–c). Diabetes mellitus ist ein Risikofaktor für die Entwicklung von Nierenzellkarzinomen. **Diabetesassoziierte Veränderungen**

Diabetesassoziierte Veränderungen

sind in einer Studie in bis zu 24 % der nierenzellkarzinombedingten Nephrektomien bei diabetischen Patienten gesehen worden [2]. Frühe Veränderungen sind glomeruläre Größenzunahme sowie Verdickung der glomerulären und tubulären Basalmembran [1, 16]. Im Verlauf treten diffuse und dann noduläre mesangiale Sklerosen (**Kimmelstiel-Wilson**

Kimmelstiel-Wilson)

auf. Es können Mesangiolysen, eine mikroaneurysmatische Dilatation der Kapillaren und hyaline Kapseltropfen in der Bowman-Kapsel entstehen. Vaskuläre Veränderungen gehören ebenfalls zur diabetischer Nephropathie, insbesondere die **Arteriolohyalinose**

Arteriolohyalinose

auch in efferenten und nicht nur afferenten Arteriolen [16]. Die Diagnose einer diabetischen Nephropathie darf nie ohne Bestätigung der Klinik, dass der Patient einen Diabetes mellitus hat, gestellt werden [16].

### Wichtige Differenzialdiagnosen

Eine gewisse mesangiale Sklerose kann auch bei **Hypertonie**

Hypertonie

vorkommen. Zudem haben Patienten mit Diabetes mellitus meist auch eine arterielle Hypertonie. Jedoch ist die Arteriolohyalinose bei hypertensiver Nephropathie häufiger nur auf die afferente Arteriole beschränkt. Eine, wenn auch nicht prominente, noduläre Glomerulosklerose kann auch bei TMA vorkommen. Sie kann sehr prominent bei **monoklonaler Immunglobulinablagerungserkrankung**

monoklonaler Immunglobulinablagerungserkrankung

(MIDD), Amyloidose, fibrillärer Glomerulonephritis, immunotaktoider Glomerulonephritis, membranoproliferativer Glomerulonephritis (MPGN) und der sog. idiopathischen nodulären Glomerulosklerose sein [1]. Letztere ist mit Rauchen und/oder Hypertonie assoziiert und wird auch „smoking-related glomerulopathy“ genannt (Abb. 4d; [17]). Die jeweiligen Diagnosen können durch zusätzliche Untersuchungen, wie Kongorotfärbung, Immunfluoreszenz oder Immunhistochemie, inkl. **Leichtkettenfärbungen**

Leichtkettenfärbungen,

Elektronenmikroskopie, und klinische Korrelation gestellt werden.

#### Renale Amyloidose

Die renale Amyloidose wird in < 1 % der Tumornephrektomien von Nierenzellkarzinomen gefunden [2, 3, 18]. In der Niere sind die **häufigsten Amyloidoseformen**

häufigsten Amyloidoseformen

Leichtketten(AL)- und Serumamyloid-A(AA)-Amyloidose, es können aber auch Schwerketten(AH)-, Apolipoprotein(AApo)-AI- und andere Apolipoproteinamyloidosen, Lysozym(ALys)-, Leukocyte-chemotactic-factor-2(ALECT2)- und Fibrinogen-α(AFib)-Amyloidosen vorkommen [19]. Es gibt unterschiedliche klinische Kontexte wie koinzidentelle AL-Amyloidose [1], systemische AA-Amyloidose bei metastasierendem Nierenzellkarzinom mit Amyloidose in der Niere und im Kolon [20] oder eine unerwartete „Bystander“-LECT2-Amyloidose [21]. Kongorotpositive Amyloidablagerungen können in der Niere die Gefäße, das Interstitium und die Glomeruli befallen. Typischerweise führen **Amyloidablagerungen**

Amyloidablagerungen

in den Glomeruli zu sog. „hahnenkammähnlichen“ Veränderungen der glomerulären Basalmembran und mesangialer Verbreiterung, die meist diffus ist, teils aber auch als nodulär imponieren und eine Differenzialdiagnose der nodulären Glomerulosklerose darstellen kann (Abb. 4e, f). Diese glomerulären Veränderungen gehen üblicherweise mit einer großen Proteinurie einher.

#### Fokal segmentale Sklerose

Eine segmentale Sklerose umschreibt die Vernarbung von < 50 % des Kapillarkonvoluts eines Glomerulus, der Begriff *fokal* besagt, dass die Läsionen < 50 % aller Glomeruli betreffen. Eine segmentale Sklerose kann als Hauptmerkmal bei der *fokal segmentalen Glomerulosklerose* (FSGS) im Sinne einer primären Erkrankung der **Podozyten**

Podozyten

vorkommen oder sekundär als Läsion bei verschiedenen chronischen Nierenerkrankungen [22]. Von der Ätiologie her werden 6 FSGS-Gruppen unterschieden [23]: Die 3 häufigsten sind die primäre FSGS, die adaptive und die APOL1-FSGS; die 3 selteneren die genetische, die medikamentenassoziierte und die virale FSGS. In Nephrektomien bei Patienten mit fortgeschrittener chronischer Nierenerkrankung handelt es sich bei der FSGS meist um eine sekundäre Form bei Hyperfiltration und **fortgeschrittener Nephrosklerose**

fortgeschrittener Nephrosklerose

[1] bzw. hypertensiver Nephropathie. Die FSGS wurde in 2–6,7 % bei Tumornephrektomien gefunden [2, 18, 24]. Andere sekundäre Formen der FSGS kommen z. B. bei Adipositas, Infektionen (HIV), medikamentenassoziiert (z. B. **Lithium**

Lithium

**Figure 5:**
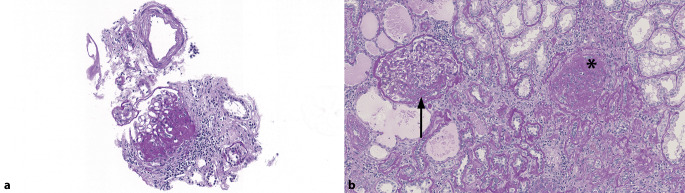

[Abb. 5a], Bisphosphonate, Kalzineurininhibitoren) und als **adaptive Veränderungen**

adaptive Veränderungen

bei Nephronmangel oder -verlust vor (z. B. Nierenhypoplasie, niedriges Geburtsgewicht, Refluxnephropathie, Anabolikaabusus, Diabetes mellitus). Der Begriff primäre FSGS wird für die Form mit primärer Schädigung der Podozyten (Podozytopathie) gebraucht, die am ehesten auf einen unbekannten zirkulierenden Faktor zurückzuführen ist. Sowohl bei genetischen als auch bei sporadischen Formen werden immer mehr **Mutationen**

Mutationen

vor allem in podozytären Genen identifiziert. Zudem finden sich bei genetischen Untersuchungen von Patienten mit FSGS immer häufiger Mutationen in Kollagen-Typ IV-Genen mit Varianten in den Genen *COL4A3, COL4A4* oder *COL4A5* [25]. Diese Mutationen sind mit einem Spektrum von **hereditären Nephropathien**

hereditären Nephropathien

assoziiert, das von mikroskopischer Hämaturie und dünner glomerulärer Basalmembran über FSGS (Abb. 5b) bis hin zum terminalen Nierenversagen und zu Endstadiumnieren reichen kann [26]. Bei der Therapie der FSGS ist insbesondere die Differenzierung einer durch eine **Podozytopathie**

Podozytopathie

induzierten primären FSGS von einer sekundären, adaptiven FSGS von großer Relevanz. Daher ist es wichtig, diese bestmöglich zu unterscheiden. Morphologische Indizien für ein sekundäres Geschehen können z. B. Zeichen einer diabetischen oder hypertensiven Nierenschädigung mit deutlichen **Parenchymvernarbungen**

Parenchymvernarbungen

oder Hinweise auf eine Glomerulonephritis sowie klinische Befunde wie eine schwere Adipositas oder Einnierigkeit sein. Bei der primären FSGS hingegen ist in den frühen Stadien häufig das Nierenparenchym kaum vernarbt und es finden sich segmentale Sklerosierungen, die häufig intrakapilläre Schaumzellen aufweisen und im Harnpolbereich gelegen sind. Während dieser Befund für eine primäre FSGS zwar nicht spezifisch ist, ist er jedoch bei entsprechender typischer klinischer Präsentation mit nephrotischer und häufig schnell aufgetretener Proteinurie sehr verdächtig auf eine primäre, podozytopathische Genese. Eine weitere Einordnung muss dann mithilfe der Elektronenmikroskopie erfolgen, bei der man einen > 75 %igen Verlust bzw. eine plattenartige Verschmelzung der Fußfortsätze der Podozyten erwarten würde. Bezüglich Beurteilung und Zuordnung einer FSGS sollte die Biopsie immer dann an eine Nephropathologie geschickt werden, wenn eine sekundäre FSGS bei offensichtlicher diabetischer oder hypertensiver Nephropathie und entsprechendem klinischem Verlauf nicht zweifelsfrei zu diagnostizieren ist.

## Tubulointerstitielles Kompartiment

*Interstitielle Fibrose und Tubulusatrophie* (IFTA) sind per se unspezifische chronische Veränderungen des tubulointerstitiellen Kompartiments. Sie können als Komponente im Verlauf verschiedener glomerulärer und vaskulärer Erkrankungen oder als primäre **tubulointerstitielle Erkrankung**

tubulointerstitielle Erkrankung

entstehen. Veränderungen im unmittelbar tumornahen Nierenparenchym (etwa 1 mm) mit IFTA und häufiger **Begleitentzündung**

**Figure 6:**
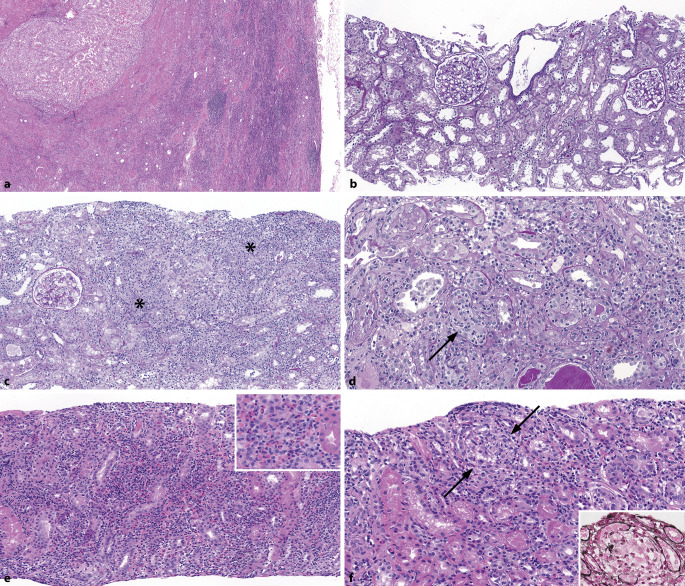

Begleitentzündung

sollten nicht als repräsentativ oder diagnostisch für eine tubulointerstitielle Nephritis interpretiert werden (Abb. 6a). Bei hypertensiver und diabetischer Nephropathie findet sich in der IFTA häufig eine interstitielle Begleitentzündung. Ein Saum von 5 mm nichtneoplastischen Parenchyms gilt für die Beurteilung als prinzipiell genügend, man sollte jedoch möglichst **tumorfernes Parenchym**

tumorfernes Parenchym

untersuchen. Eine Studie zeigte, dass bereits > 10 % IFTA zu einem signifikanten postoperativen Anstieg des Kreatininniveaus geführt haben [18].

### Medikamenteninduzierte Nephropathien

Medikamenteninduzierte Nephropathien können sehr unterschiedliche Morphologien aufweisen und alle Kompartimente betreffen. Das tubulointerstitielle Kompartiment kann mit unterschiedlich stark ausgeprägten entzündlichen Infiltraten, wie z. B. bei der chronischen **tubulointerstitiellen Nephritis**

tubulointerstitiellen Nephritis

(TIN) bei Mesalazintherapie, aber auch ohne wesentliche Entzündung vorwiegend in Form von IFTA, z. B. bei der lithiumassoziierten Nephropathie, betroffen sein. Der Nachweis von **Granulomen**

Granulomen

ohne Nekrosen bzw. eine granulomatöse TIN [27, 28, 29] oder das Vorkommen **eosinophiler Granulozyten**

eosinophiler Granulozyten

[30] kann Hinweis auf eine medikamentenassoziierte Genese sein (Abb. 6c–f). Immer mehr Patienten mit Tumoren solider Organe und hämatologischen Tumoren, so auch Patienten mit Nierenzellkarzinomen, werden mittels **Immuncheckpointinhibitoren**

Immuncheckpointinhibitoren

(ICI; z. B. PD‑1 und PD-L1-Hemmer) behandelt. Bei dieser Therapie tritt als Nebenwirkung vor allem eine akute, manchmal granulomatöse TIN auf [31, 32], aber auch Glomerulonephritiden wie Lupus-Nephritis [33] oder pauci-immune Glomerulonephritis und Vaskulitis [34]. Medikamentenassoziierte Nephropathien haben selten pathognomonische Merkmale und das verursachende Medikament muss im klinischen Kontext eruiert werden. Selten ist der morphologische Befund typisch, wie z. B. bei einer Lithiumnephropathie der Nachweis von **Zysten**

Zysten

(Sammelrohrzysten) und einer fokal segmentalen Glomerulosklerose (Abb. 5a und 6b), sodass bei einem Funktionsverlust diese Veränderungen in der Nierenbiopsie auf die spezifische medikamentöse Ursache hinweisend sein können und diese Diagnose somit die weitere Therapie beeinflussen kann. Bei klassischen Chemotherapien wird tubulointerstitiell vor allem eine akute tubuläre Schädigung gesehen. Die meisten Veränderungen sind nicht spezifisch, beim Beispiel von **Ifosfamid**

Ifosfamid

wurde aber immer wieder ein Zusammenhang mit akuten tubulointerstitiellen Schäden mit auffälligen reaktiven Zellveränderungen erwähnt [35]. Da Nierenzellkarzinome auf klassische Chemo- und Radiotherapie nicht ausreichend ansprechen, werde diese, nebst der bereits erwähnten Anti-VEGF-Therapie, vor allem mit ICI behandelt, was als Nebenwirkung vorwiegend zur TIN führen kann.

### Obstruktive Nephropathie

Die makroskopische Diagnose einer obstruktiven Nephropathie ist bei Dilatation des **Nierenbeckenkelchsystems**

**Figure 7:**
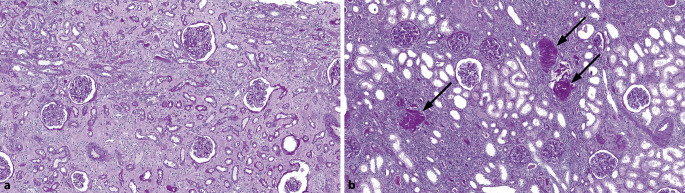

Nierenbeckenkelchsystems

einfach. Histologisch wegweisend sind eine diffuse interstitielle Fibrose und Tubulusatrophie, Tamm-Horsfall-Protein-Extravasate und -Regurgitation in den Bowman-Kapsel-Raum (Abb. 7a, b). Herdförmig betont können lymphoplasmazelluläre Infiltrate vorkommen. Prinzipiell entsprechen interstitielle entzündliche Infiltrate meist eher einer Begleitentzündung und seltener einer interstitiellen Nephritis als einer eigenständigen Erkrankung.

### Leichtketten-Cast-Nephropathie

Die Leichtketten-Cast-Nephropathie (sog. **Myelomniere**

Myelomniere)

tritt bei Plasmazellmyelomen auf [36, 37]. Klinisch ist die Diagnose manchmal klar und die Nierenbiopsie erfolgt, um die Diagnose zu sichern und das Erholungspotenzial aufgrund der Ausprägung der IFTA abzuschätzen. Manchmal ist eine Leichtketten-Cast-Nephropathien jedoch die **Erstmanifestation**

**Figure 8:**
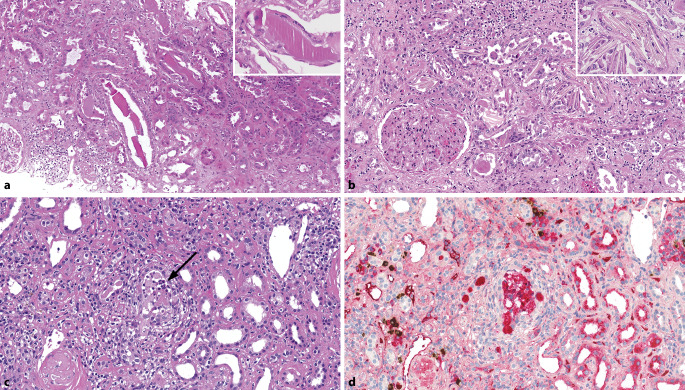

Erstmanifestation

eines Plasmazellmyeloms mit akutem Nierenversagen. Im Idealfall zeigen sich glasige, hypereosinophile **intratubuläre Proteinzylinder**

intratubuläre Proteinzylinder

(Casts) mit Bruchartefakten sowie aufgelagerten **mehrkernigen Riesenzellen**

mehrkernigen Riesenzellen

(Abb. 8a), manchmal sind sie auch nadelförmig (Abb. 8b). Häufig finden sich intratubulär **neutrophile Granulozyten**

neutrophile Granulozyten.

Manchmal sind die intratubulären Casts atypisch, feingranulär, blass, vermischt mit Neutrophilen (Abb. 8c) oder beinhalten Amyloid.

### Wichtige Differenzialdiagnosen

Ein Fallstrick in der Diagnostik der Lichtketten-Cast-Nephropathie ist die **akute Pyelonephritis**

akute Pyelonephritis,

insbesondere wenn atypische Casts mit zahlreichen Neutrophilen vermischt sind. Hilfreich ist abgesehen vom klinischen Kontext der immunhistochemische Nachweis einer **Leichtkettenrestriktion**

Leichtkettenrestriktion

in den Casts (Abb. 8d). Die Differenzialdiagnose pigmentierter intratubulärer Zylinder schließt die akute tubuläre Schädigung bei Rhabdomyolyse, Hämolyse, cholämischer Nephrose und medikamentenassoziierten Zylindern ein [38, 39, 40].

## Fazit für die Praxis

- Im Nierenparenchym können nebst zahlreichen entzündlichen und immunologischen Prozessen, die schnell therapiert werden müssen, auch zahlreiche nichtentzündliche Erkrankungen in allen Kompartimenten vorkommen, die insbesondere bei Patienten mit Nierentumoren bezüglich der Prognose ausschlaggebend sein können.
- Vaskuläre Erkrankungen, insbesondere bei Hypertonie, die diabetische Nephropathie, das Ausmaß der interstitiellen Fibrose, Tubulusatrophie und Glomerulosklerose haben prognostische Bedeutung und sollten immer im Bericht erwähnt werden.
- Das Gewebe in unmittelbarer Tumornähe (ca. 1 mm) ist nicht repräsentativ für das tumorfreie Nierenparenchym. Ein Saum von 5 mm gilt als dafür repräsentativ.
- Eine noduläre mesangiale Sklerose kann außer bei Diabetes mellitus u. a. auch bei der monoklonalen Immunglobulinablagerungserkrankung, bei Amyloidose und der sog. idiopathischen nodulären Glomerulosklerose assoziiert mit Rauchen vorkommen.
- Interstitielle entzündliche Infiltrate entsprechen meist einer Begleitentzündung und seltener einer interstitiellen Nephritis als einer eigenständigen Erkrankung.
- Die endgültige Diagnose muss in der Nierenpathologie unter Berücksichtigung der Immunhistochemie, Elektronenmikroskopie und klinischen Präsentation erfolgen.
- Zu jedem Nierentumorbefund gehört auch eine Aussage zum nichtneoplastischen Nierenparenchym, sei es auch: „kein beurteilbares nichtneoplastisches Nierengewebe“ bei einer Tumorenukleation oder „altersentsprechendes, unauffälliges Nierengewebe“.

## CME-Fragebogen

Welche Aussage zur Befundung nicht-neoplastischen Nierenparenchyms in Tumornephrektomie stimmt?

Einige Risikofaktoren für die Entstehung eines Nierenzellkarzinoms können auch das Nierenparenchym schädigen.

Arteriosklerotische Veränderungen sind ein für die weitere Nierenfunktion irrelevanter Befund.

Koinzidentielle Nierenerkrankungen sind insgesamt sehr selten.

Häufige auslösende Erkrankungen einer Nierenschädigung sind Glomerulonephritiden.

Durch Dialyse kann der Verlust einer Niere bei vorbestehender Nierenerkrankung problemlos und ohne Einfluss auf das Überleben kompensiert werden.

Welche Aussage zur Befundung von Tumornephrektomie/Tumorresektaten stimmt?

Die Befundung des nicht-neoplastischen Nierengewebes ist nach Empfehlung nur bei erhöhtem Kreatininwert integraler Bestandteil der Beurteilung von Tumornephrektomien.

Mindestens 2 cm Nierenparenchym neben dem Tumor sind für eine sinnvolle Beurteilung der Niere notwendig.

Es reicht Glomeruli und Gefäße bei der Begutachtung zu berücksichtigen.

Die direkt an den Tumor angrenzende Pseudokapsel aus komprimiertem Nierenparenchym ist nicht als repräsentativ zu betrachten.

In jedem Fall ist eine ausführliche histologische, immunhistochemische und elektronen-mikroskopische Untersuchung des Nierenparenchyms indiziert.

Welche Aussage zur hypertensiven Nephropathie ist *falsch*?

Ein interstitielles Entzündungsinfiltrat ist mit der Diagnose einer hypertensiven Nephropathie nicht vereinbar.

Globale und segmentale glomeruläre Vernarbungen kommen vor.

Tubulointerstitielle Vernarbungen sind ein häufiger Bestandteil einer hypertensiven Nephropathie.

In den Arterien findet sich eine Lamellierung der Lamina elastica interna.

Im Rahmen des Hypertonus entstehende Parenchymnarben sind häufig subkapsulär akzentuiert.

Welche Aussage zur Glomerulosklerose trifft zu?

Global sklerosierte Glomeruli sind grundsätzlich als Ausdruck einer Nierenerkrankung zu betrachten.

Im Rahmen des physiologischen Alterungsprozesses kann es zu renalen Vernarbungen kommen.

Glomeruläre Vernarbungen entstehen fast immer im Rahmen primärer glomerulärer Erkrankungen und nur in seltenen Fällen infolge einer vaskulären Erkrankung.

Bei Kindern sind bis zu 20% global sklerosiert Glomeruli noch normal.

Das Ausmaß der globalen Glomerulosklerose nimmt keinen Einfluß auf die Nierenfunktion nach chirurgischem Eingriff.

Welche Aussage in Abb. 9 dargestellten Veränderung von Glomeruli und Arteriolen trifft *nicht* zu?

Als Differenzialdiagnose ist eine monoklonale Gammopathie renaler Signifikanz zu berücksichtigen.

Die wahrscheinlichste Diagnose ist eine diabetische Nephropathie.

Nach Ausschluss eines Diabetes mellitus und anderer Ursachen käme auch eine idiopathische noduläre Glomerulosklerose in Betracht.

Die arterioläre Hyalinose im Polgefäß ist pathognomonisch für einen Diabetes mellitus.

Auch membranoproliferative Glomerulonephritiden sind in der Differenzialdiagnose zu berücksichtigen.

Bei einem 65jährigen Mann wurde aufgrund einer Angina pectoris eine Koronarangiopathie durchgeführt, worauf sich eine akute Niereninsuffizienz entwickelt hat. Welche Aussage zu dem in Abb. 10 dargestellten Befund trifft zu?

Es handelt sich um ein Fixierungsartefakt.

Die Erkrankung tritt nur im Zusammenhang mit vaskulären Eingriffen auf.

Es handelt sich um eine thrombotische Mikroangiopathie.

Man erkennt zahlreiche Cholesterinkristallspaltartefakte neben thrombotischem Material.

Es handelt sich um eine Vaskulitis mit älteren Blutungsresiduen.

Welche Aussage zur fokal-segmentalen Glomerulosklerose (FSGS) stimmt *nicht* (s. Abb. 11)?

Eine segmentale Glomerulosklerose ist zunächst ein morphologisches Schädigungsmuster und keine Erkrankung per se.

Es ist nur ein Teil des Kapillarkonvoluts vernarbt.

Sie kann Folge einer hereditären Erkrankung sein.

Man findet segmentale Sklerosen in ca. der Hälfte der Tumornephrektomien.

Eine Hyperfiltration bei Nephronverlust kann zur Ausbildung segmentaler Sklerosen im Sinne einer sekundären FSGS führen.

Welche Aussage zur renalen Amyloidose trifft zu?

Zur Diagnose ist eine Sirius-Färbung notwendig.

Eine weitere Typisierung der Amyloidose ist aufgrund der therapeutischen Implikationen wichtiger Bestandteil der Diagnose.

Transthyretin ist das häufigste Vorläuferprotein bei der renalen Amyloidose.

AA-Amyloidosen sind zumeist erblich bedingt.

Eine Amyloidose ist bei nodulärer mesangialer Verbreiterung so gut wie ausgeschlossen.

Eine 70jährige Patientin wird mit akutem Nierenversagen beim Hausarzt vorstellig. Die Patientin klagt über seit längerem bestehende Rückenschmerzen. Die Urinanalyse zeigt eine hohe Proteinurie mit nur geringer Albuminurie. Welche Aussage zu dem in Abb. 12 dargestellten Befund ihrer Nierenbiopsie trifft zu?

Es handelt sich um eine interstitielle Nephritis mit intratubulären Konkrementen.

Die intratubulären neutrophilen Granulozyten sprechen für einen aszendierenden Infekt.

Auslöser der Befunde ist ein multiples Myelom.

Der Befund ist rein morphologisch keinem spezifischen Auslöser zuzuordnen.

Es dürfte sich in 1. Linie um die Begleitreaktion einer glomerulären Nierenerkrankung handeln.

Welche Aussage zur interstitiellen Fibrose und Tubulusatrophie (IFTA) trifft *nicht* zu?

Der Befund ist eine chronische, fibrosierende tubulo-interstitielle Veränderung, welche mannigfaltige Ursachen haben kann.

Das direkt an den Tumor angrenzende Nierenparenchym ist hinsichtlich des Ausmaßes der IFTA nicht aussagekräftig, und es soll möglichst tumorfernes Nierenparenchym begutachtet werden.

Das Ausmaß der IFTA ist ein wichtiger prognostischer Marker der postoperativen Nierenfunktion.

Primär glomeruläre Erkrankungen gehen in typischerweise nicht mit einer IFTA einher.

Medikamente können Auslöser einer IFTA sein.
